# Can cash break the cycle of educational risks for young children in high HIV–affected communities? A cross–sectional study in South Africa and Malawi

**DOI:** 10.7189/jogh.07.020409

**Published:** 2017-12

**Authors:** Lorraine Sherr, Mark Tomlinson, Ana Macedo, Sarah Skeen, Imca Sifra Hensels, Lucie Dale Cluver

**Affiliations:** 1 Research Department of Global Health, University College London, London, United Kingdom; 2 Department of Psychology, Stellenbosch University, Stellenbosch, South Africa; 3 Department of Psychiatry and Mental Health, University of Cape Town, Cape town, South Africa; 4 Department of Psychology, University of Manchester, Manchester, United Kingdom; 5 Centre for Evidence–Based Intervention, Department of Social Policy & Social Intervention, University of Oxford, Oxford, United Kingdom

## Abstract

**Background:**

Household cash grants are associated with beneficial outcomes; enhanced if provided in combination with care.

**Objectives:**

This study describes the impact of cash grants and parenting quality on 854 children aged 5–15 (South African and Malawi) on educational outcomes including enrolment, regular attendance, correct class for age and school progress (controlling for cognitive performance). Consecutive attenders at randomly selected Community based organisations were recruited. The effects of cash plus good parenting, HIV status and gender were examined.

**Results:**

Overall 73.1% received a grant – significantly less children with HIV (57.3% vs 75.6% (χ^2^ = 17.21, *P* < 0.001). Controlling for cognitive ability, grant receipt was associated with higher odds of being in the correct grade (odds ratio (OR) = 2.00; 95% confidence interval (CI) = 1.36, 2.95), higher odds of attending school regularly (OR = 3.62; 95% CI = 1.77, 7.40), and much higher odds of having missed less than a week of school recently (OR = 8.95; 95% CI = 2.27, 35.23). Grant receipt was not associated with how well children performed in school compared to their classmates or with school enrolment. Linear regression revealed that grant receipt was associated with a significant reduction in educational risk (B = –0.32, *t*(420) = 2.84, *P* = 0.005) for girls.

**Conclusion:**

Cash plus good parenting affected some educational outcomes in a stepwise manner, but did not provide additive protection.

Cash transfers are an effective intervention to enhance child outcomes in a number of studies [1,2]. Cash transfer initiatives were first considered in areas of poverty and deprivation, mainly in South America [3,4]. The concept was based on the premise that a regular small cash allowance to families would enhance specific child development outcomes such as immunisation, birth certification, educational and health outcomes [5,6]. This was mediated by the provision of parenting programmes, health programmes and in some instances linking the cash allowance to conditions, such as requiring parents to obtain birth certification and immunisation of the child. The early evaluation literature was concerned with the efficacy of conditional vs unconditional payments. Both were found to be effective, and both had beneficial child developmental outcomes [7]. The challenge with the conditional approach was that the conditions had the benefit of prompting uptake of important behaviours, but a sanctioning or punitive approach to the most vulnerable families when parents were unable to meet the conditions seemed counterproductive. This then evolved to the provision of alternative supports (such as cash buddies) for defaulting groups. Other studies showed that if grants were made non–conditional, the majority of recipients used the cash for the benefit of the child anyway. A recent review and meta–analysis of financial incentives found good evidence that they could promote coverage of several important child health interventions [8].

The HIV/AIDS epidemic brought a further set of child health challenges resulting from illness, death, orphaning and poverty. In addition, a number of HIV–related behaviours emerged that needed prompting, such as HIV testing, medication adherence and clinic attendance. In widely–affected environments of parental and child HIV infection, economic and social challenge and daily poverty, this evidence–based intervention was considered worthy of adaptation to African HIV settings [1,9]. Early studies in Africa explored the benefits of conditional vs unconditional provision [10]. Some studies went further to use cash incentives to promote HIV–avoidance behaviours. Subsequent studies in various countries were set up to examine the efficacy of large cash transfer programmes – in whatever form – on child outcome [11]. In South Africa, an unconditional child grant programme was initiated in the late 90s, as part of a range of social security reforms post–apartheid, and has been found to have a positive impact on a range of child outcomes [12]. As a result, cash grants have improved dramatically in terms of availability in the region, with increasing roll–out of old age pensions and child–focused grants [13,14]. Cash transfers were explored as an effective intervention to reduce risk behaviour, to enhance HIV testing, to remain HIV and STI free [15–17]. Most of the trials have concentrated on adolescent behaviour and outcome s[18] or explored the more cash incentive components[8]. Outside of trials, it was important to monitor the impact of cash grants or cash injections on both direct recipients and wider family or household members [19]. This was particularly relevant to children within the recipient households. Studies using large population samples examined adolescent HIV risk behaviour and showed that cash transfers – the receipt of any regular cash allowance into a family – had specific effects on reducing adolescent girls’ HIV risk behavior [20,21]. However, this study showed that there was no similar significant effect on boys. A subsequent analysis explored the broader needs and showed that cash plus psychosocial care not only reduced female HIV risk behaviours more than cash alone, but cash plus care had a significant effect on reducing HIV risk behaviour amongst boys. To date the literature clearly shows that cash transfers within studies and within broader government provision is an effective intervention for child outcomes. Furthermore, it is clear that cash alone is not a magic bullet and there are some groups and some outcomes that are not reached [22]; enhancing provision by looking at a more comprehensive cash plus care shows the additive effects and wider impact when cash is part of a more robust approach to supporting children [23].

This focus on adolescence and adolescent risk behaviours within the HIV setting is important, but tells us little about the effects of cash transfers on the younger child. It is important to understand the effects of early commencement of cash support, and whether receipt could break the cycle of predictions leading to HIV risk behaviours in the first place. A particularly well–established pathway to HIV–risk behaviours is educational failure and/or drop out. Two reviews and subsequent studies have shown that educational attendance and achievement are protective against HIV risk behaviours [24–27]. A recent study [28] examined the risk behaviour of age disparate sex and noted that school enrolment was associated with lower adolescent pregnancy rates. Another recent study showed path models between poor educational outcomes and familial HIV/AIDS, poverty, child internalising problems and gender. The four educational measures included enrolment, attendance, grade progression and concentration [29]. A trial in Malawi [30] showed that a cash transfer programme for adolescent girls based on schooling showed a reduction in HIV and Herpes Simplex, and two new randomised trials in South Africa have both shown protective effects of education on adolescent girls’ HIV risk [31,32].

It thus seems important to examine the effects of cash transfer programmes on educational outcomes for younger children – prior to the age of HIV associated risk behaviours – and to examine this essential precursor of risk in an attempt to break the risk cycle. This study was therefore set up to explore the effects of cash grants on educational outcomes in younger children, aged 5–15. The study was set up to examine whether cash grants divert poor educational outcomes for children and thus may serve to interrupt the pathways to risk in later adolescence. If such a relationship between cash and educational outcomes exists, a further analysis would be helpful to see if care, defined as good parenting, enhanced or magnified cash effects on education for both girls and boys. In the context of known cognitive delays related to paediatric HIV–infection, uninfected exposed children [33,34] and poverty, it is also essential to examine whether any positive associations of cash and care on educational outcomes are independent of cognitive ability and delay. On top of that, it would be worthwhile to see whether the effects of cash and/or care on educational outcomes differ by child HIV status.

## METHODS

### Participants

The data from this study emerged from the longitudinal Child Community Care study which took place in 2013–2014, tracking the development of children infected and affected by HIV/AIDS who attend community–based organisations (CBOs) in South Africa and Malawi. The CBOs in question were selected from a list composed of all CBOs (n = 588) funded by 11 funding partners (World Vision, Comic Relief, Save the Children, Firelight Foundation, Help Age, UNICEF, REPSSI, Bernard van Leer Foundation, STOP AIDS Now, AIDS Alliance and the Diana Memorial Fund). All 588 CBOs were stratified by funder and 28 were randomly selected, prorated for population size, resulting in 28 CBOs in South Africa and four in Malawi. Ethical approval was obtained from the ethics boards of University College London (reference number 1478/002) and Stellenbosch University (reference number N10/04/112).

All CBO’s consented to inclusion in the study. All caregivers received full information outlining the study and clarifying the voluntary nature of participation, the consent procedures for themselves and their child, the confidentiality around the study and the ability to withdraw at any time with no consequences. Caregiver consent was provided in writing and with the process provided orally and on written information sheets for them to keep. In addition assent was gained from all children with standardised and age appropriate information explained. Participation was voluntary and was not paid. Children were provided with a fruit snack and a drink together with a participation certificate, while caregivers were provided with a small food/grocery item. Child protection issues were handled with a full referral procedure in place if required or requested to CBO and local health/social services.

### Procedure

Consecutive children between the ages of 5–15 years old and their primary caregivers were interviewed by thoroughly trained data collectors using mobile phone technology [35]. The questionnaires were translated and back–translated into Zulu and Xhosa and the participants were interviewed in the language of their choice. Data were collected in 2013–2014. Participation included 854 children and their primary caregiver.

### Measures

**Child HIV status** was determined using caregiver report. Children were classified either as confirmed HIV–positive or as non–HIV–positive, with the latter comprising both children who were confirmed HIV–negative and children who had never been tested. Caregiver HIV status was assessed using self–report. **Educational outcomes** were assessed using questions from the Child Status Index (CSI) tool [36]. Caregivers were asked to report on children’s enrolment in school, access and learning outcomes. This comprised six items: 1) school enrolment (“Is your child enrolled in school?” Response categories were *yes* or *no*); 2) school regular attendance (“Does your child go to school?” Response categories were *yes regularly, yes sometimes, yes but rarely*, or *no*); 3) school non–attendance (“How many school days did the child miss in the past two weeks?” Responses were coded as whether the child missed school days for an extended period (>1 week) during the previous weeks); 4) being in the age–appropriate school grade (“Is your child in the correct class for his or her age?” Response categories were *yes* or *no*); 5) school performance (“How do teachers report your child is doing in school?” Response categories were *he or she does better than most children, he or she does as well as other children,* or *he or she struggles at school*), and 6) learning progress (“Is the child quick to learn when introduced to new chores and things?” Response categories were *yes, no* or *don’t know*). All six items were converted into a binary (yes/no) variable. **Educational risk** was a composite measure made up of five binary variables. This included incorrect school grade for age, irregular school attendance, rated as a slow learner, reported as struggling in school, and missing more than a week of school recently. For each affirmative answer to these five questions, the child’s educational risk score increased by one, resulting in a total score ranging from 0–5 – with the higher score indicating higher educational risk. **Grant receipt** (any/none) was determined based on whether the caregivers reported receiving one or more of the following grants into the home: a retirement pension, state pension, disability grant, child support grant, foster child grant, or care dependency grant. **Number of grants** was the number of grants received in total by a family (range from 0–6). **General cognitive ability** was measured using the draw–a–person task [37,38]. **Good parenting** was operationalized using a composite measure of 10 items (scored by caregiver and child) related to positive parenting, affection and praise, positive discipline tactics, and lack of abuse or violence. Each item was coded as binary and a total score was calculated by summing up the ten items for a scale with a range of 0–10. Scores equal to or above 8 were then coded as good parenting and scores below 8 were coded as not good parenting [39].

### Statistical analysis

The statistical analysis comprised five steps. First, *t–*–tests and chi–square tests were run to look at differences between those receiving grants and those not receiving grants on educational variables. Second, associations between grant receipt and educational variables were tested using logistic regressions for binary variables and linear regression for continuous variables. These were also carried out split by gender. Third, linear and logistic regressions were conducted to test for interaction effects of gender and grant receipt on educational outcomes to explore whether gender was a moderating factor. Fourthly, the same series of linear and logistic regression analyses were carried out, but with an interaction between grant receipt and child HIV status included to observe whether child HIV status was a moderating factor on the associations between grant receipt and educational outcomes. Lastly, a series of regression models were used to examine associations of cash only, good parenting care only, and both cash and good parenting (represented by dummy variables, taking “neither” as the reference category) with educational outcomes. The analyses in the second, third, and fourth steps were all adjusted for general cognitive ability and age. Steps two and four were also adjusted for gender except in cases where gender was looked at as the predictor of interest (ie, in step two the analysis of the total sample is adjusted for gender, but not the analyses that are split by gender). All regression analyses were carried out separately for each educational outcome.

## RESULTS

In total, 854 children (52.3% girls; aged 5–15, *M* = 10.21, SD = 2.80) and their caregivers were interviewed (total participant N = 1708) (**Table 1**). There were 116 children (13.9%) reported as HIV+ve by their caregiver. Of the 808 caregivers, 160 (19.8%) were themselves HIV+ve. Of the 854 children, 46% were cared for by their biological parent/s and 54% by other caregivers (grandparents 28.7%). The caregivers of 73.1% of all children reported receiving at least one cash grant, and 26.9% reported receiving no grant whatsoever. Grant receipt did not differ according to child gender (caregivers of 72.3% of the boys and 73.3% of the girls received a grant; χ^2^_1_ = 0.13, *P* = 0.72). Grant receipt did differ significantly according to child HIV status, with 57.3% of the HIV–positive children receiving a grant compared to 75.6% of the non–HIV–positive children (χ^2^_1_ = 17.21, *P* < 0.001).

**Table 1:** Cross–sectional differences between those receiving cash transfer and those not receiving cash transfer on educational outcomes, split by child HIV status*

	Cash grant (n = 624)			No cash grant (n = 230)			Difference statistic (*P*–value)
	**Total**	**HIV–positive children (n = 69)**	**Non–HIV–positive children (n = 557)**	**Total**	**HIV–positive children (n = 46)**	**Non–HIV–positive children (n = 180)**	
**Educational risk**	**0.71 (1.01)**	1.22 (1.27)	0.65 (0.95)‡	**1.11 (1.23)**	1.26 (0.98)	1.07 (1.28) ‡	**4.28 (<0.001)**
**Enrolled in school**	**620 (99.4%)**	69 (100%)	551 (99.3%)†	**222 (96.5%)**	45 (97.8%)	177 (96.2%)†	**9.77 (0.002)**
**Correct class for age**	**468 (75.5%)**	38 (55.1%)	430 (78.0%)‡	**107 (48.2%)**	18 (40.0%)	89 (50.3%)‡	**56.20 (<0.001)**
**Regular attendance**	**601 (96.9%)**	63 (91.3%)	538 (97.6%)‡	**198 (89.2%)**	40 (88.9%)	158 (89.3%)‡	**20.24 (<0.001)**
**Quick learner**	443 (72.6%)	42 (61.8%)	401 (74.0%)	164 (74.5%)	28 (65.1%)	136 (76.8%)	0.30 (0.58)
**Doing as well or better than most in school**	522 (84.2%)	47 (68.1%)	475 (86.2%)	183 (82.4%)	37 (82.2%)	146 (82.5%)	0.37 (0.54)
**Missed less than a week of school**	**616 (99.4%)**	69 (100%)	547 (99.3%)‡	**210 (94.6%)**	44 (97.8%)	166 (93.8%)‡	**19.87 (<0.001)**
**Working memory**	**9.34 (3.54)**	8.25 (3.78)	9.47 (3.49) ‡	**7.98 (3.44)**	7.16 (3.50)	8.19 (3.41) ‡	**4.93 (<0.001)**
**General cognitive ability**	**95.29 (14.92)**	92.74 (15.05)‡	95.60 (14.89) ‡	**80.34 (18.47)**	69.62 (19.16) ‡	83.00 (17.34) ‡	**10.93 (<0.001)**

*Symbols denote differences on educational outcomes between HIV–positive and HIV–negative children within their grant category (ie, HIV–positive children whose caregivers receive a grant are compared with HIV–positive children whose caregivers do not receive a grant, and non–HIV–positive children whose caregivers receive a grant are compared with non–HIV–positive children whose caregivers do not receive a grant). The difference statistic (*P*–value) denotes the differences between children receiving a grant and not receiving a grant, regardless of child HIV status. Bolded variables differed significantly in the total sample according to grant receipt. Numbers occasionally do not add up to the total due to missing data.

† *P* < 0.01.

‡*P* < 0.001.

### Associations between grant receipt and educational risk

Independent of general cognitive ability, grant receipt was associated with a number of beneficial educational outcomes. As can be seen in **Table 2**, children in households receiving grants within the past year had higher odds of being in the correct grade for their age (OR = 2.00; CI = 1.36, 2.95), higher odds of attending school regularly (odds ratio (OR) = 3.62; 95% confidence interval (CI) = 1.77, 7.40), and much higher odds of having missed less than a week of school recently (OR = 8.95; 95% CI = 2.27, 35.23). However, these children also had significantly lower odds of being quick learners (OR = 0.62; 95% CI = 0.42, 0.93). Grant receipt was not associated with how well children performed in school compared to their classmates or with school enrolment in the total sample. Some of these findings were gender–specific; while the findings on attendance were comparable for boys and girls, only girls had a positive association between grant receipt and being in the correct grade (OR = 2.52; 95% CI = 1.42, 4.44). Furthermore, only for boys grant receipt was associated with lower odds of being a quick learner (OR = 0.48; 95% CI = 0.28, 0.83) and lower odds of doing as well or better than most in school (OR = 0.51; 95% CI = 0.27, 0.96). Finally, a linear regression revealed that grant receipt was not significantly associated with total educational risk for the entire sample (B = –0.14, *t*(800) = 1.55, *P* = 0.12), and also not for boys specifically (B = 0.028, *t*(377) = 0.20, *P* = 0.84). However, for girls, grant receipt was associated with a significant reduction in educational risk (B = –0.32, *t*(420) = 2.84, *P* = 0.005).

**Table 2:** Cross–sectional logistic regression outcomes of receiving cash transfer (0 = no, 1 = yes), or cash plus care (0 = no cash and no care, 1 = either cash or care, 2 = cash plus care) on educational outcomes*

	Model 1: OR (95% CI)			Model 2: OR (95% CI)		
	**Total**	**Boys**	**Girls**	**Total**	**Boys**	**Girls**
**Enrolled in school**	4.27 (0.94, 19.41)	9.48 (0.87, 103.53)	2.07 (0.29, 14.85)	1.72 (0.55, 5.40)	4.00 (0.57, 28.37)	1.01 (0.24, 4.24)
**Correct class for age**	2.00 (1.36, 2.95)§	1.58 (0.93, 2.68)	2.52 (1.42, 4.44)‡	1.31 (0.98, 1.74)	1.04 (0.70, 1.55)	1.64 (1.08, 2.51)†
**Regular attendance**	3.62 (1.77, 7.40)§	4.27 (1.53, 11.93)‡	3.18 (1.15, 8.83)†	1.97 (1.10, 3.52)†	2.13 (0.91, 5.03)	1.85 (0.84, 4.11)
**Quick learner**	0.62 (0.42, 0.93)†	0.48 (0.28, 0.83)‡	0.90 (0.49, 1.65)	0.84 (0.63, 1.10)	0.61 (0.42, 0.90)†	1.21 (0.80, 1.81)
**Doing as well or better than most in school**	0.70 (0.44, 1.14)	0.51 (0.27, 0.96)†	1.20 (0.56, 2.57)	0.92 (0.66, 1.29)	0.72 (0.46, 1.12)	1.32 (0.77, 2.28)
**Missed less than a week of school**	8.95 (2.27, 35.23)‡	10.81 (1.17, 100.30)†	8.37 (1.42, 49.26)†	3.95 (1.38, 11.32)†	5.29 (0.96, 29.03)	3.28 (0.84, 12.78)

OR – odds ratio, CI – confidence interval

*Model 1 is effect of receiving a cash transfer adjusted for general cognitive ability, child age and child gender. Model 2 is the effect of receiving cash plus care, either or neither, adjusted for the same covariates. Analyses for all educational outcomes were done separately.

†*P* < 0.05.

‡*P* < 0.01.

§*P* < 0.001.

### Moderation analysis: Gender

**Figure 1:**
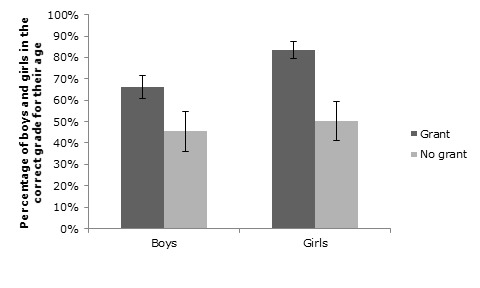
Moderation effect of gender on the association between grant receipt and being in the correct grade for age (odds ratio (OR) = 1.97; 95% confidence interval (CI) = 1.01, 3.84; *P* = 0.048).

Moderation analyses for an interaction effect between child gender and grant receipt on educational outcomes only uncovered an interaction effect on the child being in the correct grade for their age (OR = 1.97; 95% CI = 1.01, 3.84; **Figure 1**). This shows that while for both genders the odds of being in the correct grade for their age improved somewhat as a result of grant receipt, the improvement of girls as a result of cash transfer was significantly larger than the improvement of boys. 50.4% of the girls whose households did not receive a grant were in the correct grade compared to 83.4% of the girls whose households did receive a grant. For the boys, 45.4% of those not receiving grants were in the correct class, compared to 66.3% of those who did receive a grant. On the other educational outcomes, no moderation effect of gender was found.

### Moderation analysis: Child HIV status

**Figure 2:**
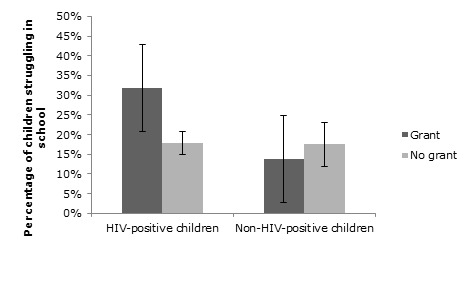
Moderation effect of child HIV status on the association between grant receipt and school performance (odds ratio (OR) = 0.34; 95% confidence interval CI = 0.11, 1.004; *P* = 0.051).

A marginally significant moderation effect of child HIV status (**Figure 2**) was found on the association between receiving a grant and struggling in school (OR = 0.34; 95% CI = 0.11, 1.004, *P* = 0.051), on top of the main effects of child HIV (OR = 8.64; 95% CI = 1.91, 39.01, *P* = 0.005) and grant receipt (OR = 0.10; 95% CI = 0.01, 0.74, *P* = 0.024). Upon further inspection, this was due to the fact that even though within the non–HIV–positive children those receiving and not receiving a grant have similar proportions of children struggling in school (13.8% and 17.5% respectively), among the HIV–positive children receiving a grant was associated with much more struggling in school (31.9%) than among the children whose caregivers do not receive a grant (17.8%). On other educational outcomes (school attendance, educational risk, being in the correct class for age, being a quick learner, and having missed school for more than a week recently), no moderation effect of child HIV was found. A moderation analysis on school enrolment could not be performed because all HIV–positive children were enrolled in school.

### Associations between grant receipt (cash) and good parenting (care) on educational outcomes

Of the total, 21.0% of the children (n = 179) did not receive cash or good care, 61.4% of the children (n = 524) received either cash or good care, and 17.7% of the children (n = 151) received both cash and care. In a linear regression model, receiving cash, care or a combination of both was not associated with educational risk for the total sample (B = –0.068, t(800) = 1.10, *P* = 0.27), or for boys (B = 0.086, *t*(377) = 0.85, *P* = 0.39). For girls, however, receiving cash, care, or a combination of both was associated with a reduction in educational risk in a stepwise manner (B = –0.20, *t*(420) = 2.66, *P* = 0.008).

As can be seen in **Table 2**, cash or care and cash plus care was not associated with most of the other education outcomes. However, it was associated in a stepwise manner with higher odds of regular attendance in the total sample (OR = 1.97; 95% CI = 1.10, 3.52) and higher odds of having missed less than a week of school (OR = 3.95; 95% CI = 1.38, 11.32). For girls (OR = 1.64; 95% CI = 1.08, 2.51) but not for boys (OR = 1.04; 95% CI = 0.70, 1.55) cash and/or care was associated with higher odds of being in the correct grade for age. For boys (OR = 0.61; 95% CI = 0.42, 0.90) but not girls (OR = 1.21; 95% CI = 0.80, 1.81) it was associated with lower odds of being a quick learner.

Upon further inspection, only the step from receiving no cash and no care to receiving either cash or care was associated with a significant improvement in educational outcomes (**Table 3**).

**Table 3:** Cross–sectional one–way ANOVA and chi–square results of receiving no cash or care, either cash or care, or cash plus care on cognitive abilities and educational risk*

	Boys (n = 400)						Girls (n = 439)					
	**No cash, no care (A)**	**Cash or care** **(B)**	**Cash plus care (C)**	**Difference statistic (*P*–value)**	**Post–hoc A vs B (95% CI)**	**Post–hoc B vs C (95% CI)**	**No cash, no care (A)**	**Cash or care** **(B)**	**Cash plus care (C)**	**Difference statistic (*P*–value)**	**Post–hoc A vs B (95% CI)**	**Post–hoc B vs C (95% CI)**
**Educational risk**	1.21 (1.38)	1.01 (1.15)	0.92 (1.06)	1.22 (0.30)	0.20 (–0.16, 0.56)	0.09 (–0.32, 0.50)	1.03 (1.10)	0.55 (0.93)	0.41 (0.70)	11.59 (<0.001)	0.49 (0.21, 0.76)‡	0.14 (–0.14, 0.42)
**Correct class for age**	39 (47.0%)	161 (63.9%)	40 (65.6%)	5.23 (0.016)	7.41‡	0.061	44 (50.0%)	208 (79.7%)	71 (85.5%)	37.08 (<0.001)	28.91‡	1.41
**Regular attendance**	73 (88.0%)	244 (96.8%)	59 (96.7%)	10.72 (0.005)	9.67†	0.002	79 (89.8%)	254 (97.3%)	79 (95.2%)	8.49 (0.014)	8.57†	0.93
**Quick learner**	58 (70.7%)	159 (64.1%)	38 (63.3%)	1.33 (0.52)	1.20	0.013	67 (77.0%)	202 (78.6%)	71 (85.5%)	2.32 (0.32)	0.10	1.91
**Doing well or better than most in school**	65 (78.3%)	190 (75.4%)	51 (83.6%)	1.95 (0.38)	0.29	1.87	76 (86.4%)	234 (89.7%)	78 (94.0%)	2.72 (0.26)	0.72	1.39

CI – confidence interval

*Difference statistic is F for continuous variables and χ2 for categorical variables. Post–hoc comparisons are mean difference (odds ratio) between categories for continuous variables (ie, A–B and B–C) and χ2 for categorical variables. Because half of the cells in school enrolment and school days missed had fewer than five cases, a chi–square analysis for these variables split by gender could not be carried out.

†*P* < 0.01.

‡*P* < 0.001.

**Figure 3:**
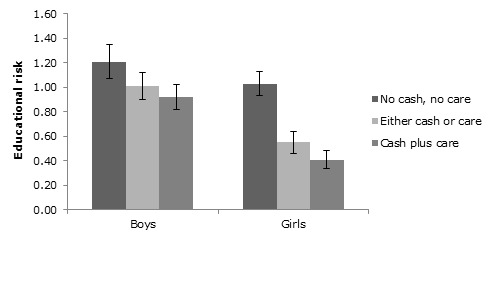
While for boys receiving either cash or care was not associated with significantly lower educational risk than receiving no cash and no care (*t*(328) = 1.29, *P* = 0.20), this difference was significant for girls (*t*(342) = 4.02, *P* < 0.001). For neither gender receiving cash plus care was better than receiving either cash or care.

For both boys and girls receiving cash or care was associated with a higher likelihood of being in the correct class for their age (χ^2^_1_ = 7.41, *P* < 0.01 and χ^2^_1_ = 28.91, *P* < 0.001, respectively) and a higher likelihood of regular attendance (χ^2^_1_ = 9.67, *P* < 0.01 and χ^2^_1_ = 8.57, *P* < 0.01, respectively). On top of that, for girls but not for boys receiving cash or care was also associated with lower educational risk (mean (*M*) = 0.55, standard deviation (SD) = 0.93) compared to not receiving either (*M* = 1.03, SD = 1.10; *t*(342) = 4.02, *P* < 0.001; **Figure 3**). Receiving cash plus care as opposed to only cash or only care, however, was not associated with statistically better educational outcomes for either of the two genders.

## DISCUSSION

This analysis has several implications for understanding the role of both cash grants and care provision for young children. First, our data show that receiving a grant was associated with beneficial educational outcomes: higher odds of being in the correct class for age, higher odds of regular school attendance, and higher odds of having missed less than a week of school recently. This is despite the fact that primary education in both countries studied is provided free of charge and enrolment for the overall sample was high. Previous South African research on the impact of the child support grant in particular on educational outcomes has found a positive impact on enrolment. Samson et al. [40] found increased school attendance among beneficiaries, while a study looking specifically at adolescent recipients were more significantly more likely to be enrolled than those not receiving the grant, after controlling for a range of factors [41]. However, our data show that the impact may extend further than enrolment only, which is important given that children may be enrolled but not allowed to attend because they have not been able to pay even small required fees or extra costs associated with attendance. We also found that receiving a grant was also associated with lower odds of being a quick learner. This may be a cause of grant receipt rather than a consequence; it might be that children who display symptoms of cognitive delay or disability are more likely to receive a grant in the first place and indeed may have higher needs.

Second, our data show different levels of access based on child characteristics. The HIV–positive children were less likely to receive a grant, and those who received a grant were more likely to struggle in school, perhaps also reflecting that the most needy (ie, the ones who struggle in school) more often receive a grant or come to the attention of those referring or processing grants. It may be that particular grants linked to disability are within the eligibility for some children with HIV. Indeed this is in line with findings from a study in Zimbabwe which showed that orphaned children were at a higher risk of poor social protection outcomes [19]. This finding highlights the particular needs of families affected by HIV who should be more likely to receive grants – but were less likely in our study. It may be that illness or a distraction of focus actually overlooks this need. A clear learning imperative is for clinics to check and streamline grant access for HIV positive children.

Our data also point to some consistent variation by gender, which suggests the importance of disaggregating the data by gender and perhaps considering provision with a gender focus. There were some differences between boys and girls in our findings. While the educational outcomes for both genders was associated with a significant improvement for those in receipt of a cash grant, girls improved more than boys on the variable related to correct grade for age. Also while receiving either cash or good parenting was associated with significantly lower overall educational risk for girls, this was not the case for boys. Similar variations in educational achievement outcomes associated with grant receipt have been noted in previous research on the South African grant system, with the child support grant associated with better attendance for boys in particular, and higher grade attainment, for girls [12].

Unlike the literature on HIV risk behaviour for older adolescence, our younger sample showed that receiving cash plus care did not improve educational outcomes over and above receiving either cash or care. This was the case for both girls and boys with comparable results for both genders. It may be that either is associated with educational outcomes or that with younger children the additive effect is not yet obvious. No measure on the quality of the educational experience was gathered in this study and the next step in provision may be to ensure high quality education for those who do attend and not simply count enrolment and attendance as sufficient. Perhaps the care components in this study, which were measured as good parenting, are important in their own right for educational outcomes. Other care variables utilised in adolescent studies are not necessarily relevant or available to younger children who may not, for example be able to attend support groups independently.

The study is not without limitations. This study is carried out within a community setting, in organisations that serve the most vulnerable children [42]. The respondents were gathered from CBO attenders and thus it is difficult to say if the sample is representative of the larger community. It is also possible that these organisations were facilitating both cash grant access and care provision. This needs to be taken into consideration when generalising the findings to the wider community. Despite this, grant access was not universal with approximately three quarters of caregivers receiving a grant. As the data was cross–sectional there is no way of determining causal pathways but can only demonstrate associations for further investigation. Longitudinal data would be essential to track early educational benefits into adolescence with the possibility of assessing impacts on HIV risk behaviour in the first place and ultimately sexually transmitted infections and HIV incidence. HIV status was established using caregiver report and was not confirmed using biological tests. Such reporting has been used in many studies, but without confirmatory tests it is possible that the HIV status was underestimated from those who did not wish to disclose. Educational measures would have been strengthened with teacher or school reports. In addition, it would be of value for future research to examine impacts of social protection on educational achievement for children of different ages (from early childhood development to tertiary education) and in different schooling environments. Good parenting was the only domain used to determine care. Using a more comprehensive model of care (care in the home, care at school, and care in the community) or broader parenting competencies such as skill building, might lead to different results, but no such broader care variables were gathered.

Yet, despite these limitations, these data clearly show the advantages of cash grants for a number of educational outcomes. Educational risk is a pathway for future behavioural risk, and it appears that grants may be associated with improved educational outcomes and may well interrupt the causal cycle seen in later adolescence. In other areas of child development there is clear evidence that early intervention is beneficial and that prevention of a problem is better than having to undo a problem once it is entrenched. This data thus lends support for the importance of both cash grants and good parenting defined as care on educational pathways for young children, as an investment in its own right, but also as an investment for their future. For HIV positive children, access to grants may be specifically beneficial, yet they are less likely to receive these. Integrated clinic provision may well enhance child development by including referral for grants within the package of services offered. Special attention to school and educational factors is needed for HIV positive children who may struggle academically. Many of the early conditional cash transfer studies used school enrolment as a condition. Our data suggests that in countries with universal provision of primary school, attention needs to focus on more detailed educational variables including attendance, progress and achievement. Cash grants as well as care appear to be associated with benefits on these educational outcomes for vulnerable children.
